# Pretreatment Cranial Computed Tomography Perfusion Predicts Dynamic Cerebral Autoregulation Changes in Acute Hemispheric Stroke Patients Having Undergone Recanalizing Therapy: A Retrospective Study

**DOI:** 10.3390/neurolint16060119

**Published:** 2024-11-25

**Authors:** Lehel-Barna Lakatos, Manuel Bolognese, Mareike Österreich, Martin Müller, Grzegorz Marek Karwacki

**Affiliations:** 1 Department of Neurology and Neurorehabilitation, Section Neuroradiology, Lucerne Cantonal Hospital, 6000 Lucerne, Switzerlandmanuel.bolognese@luks.ch (M.B.); mareike.oesterreich@luks.ch (M.Ö.); 2 Department of Radiology, Section Neuroradiology, Lucerne Cantonal Hospital, 6000 Lucerne, Switzerland

**Keywords:** stroke, recanalizing therapy, cerebral autoregulation, computed tomography perfusion, transfer function

## Abstract

Objectives: Blood pressure (BP) management is challenging in patients with acute ischemic supratentorial stroke undergoing recanalization therapy due to the lack of established guidelines. Assessing dynamic cerebral autoregulation (dCA) may address this need, as it is a bedside technique that evaluates the transfer function phase in the very low-frequency (VLF) range (0.02–0.07 Hz) between BP and cerebral blood flow velocity (CBFV) in the middle cerebral artery. This phase is a prognostically relevant parameter, with lower values associated with poorer outcomes. This study aimed to evaluate whether early cranial computed tomography perfusion (CTP) can predict this parameter. Methods: In this retrospective study, 165 consecutive patients with hemispheric strokes who underwent recanalizing therapy were included (median age: 73 years; interquartile range (IQR) 60–80; women: 43 (26%)). The cohort comprised 91 patients treated with intravenous thrombolysis (IV-lysis) alone (median National Institute of Health Stroke Scale (NIHSS) score: 5; IQR 3–7) and 74 patients treated with mechanical thrombectomy (median NIHSS: 15; IQR 9–18). Regression analysis was performed to assess the relationship between pretreatment CTP-derived ischemic penumbra and core stroke volumes and the dCA VLF phase, as well as CBFV assessed within the first 72 h post-stroke event. Results: Pretreatment penumbra volume was a significant predictor of the VLF phase (adjusted r^2^ = 0.040; *β* = −0.001, 95% confidence interval (CI): −0.0018 to −0.0002, *p* = 0.02). Core infarct volume was a stronger predictor of CBFV (adjusted r^2^ = 0.082; *β* = 0.205, 95% CI: 0.0968–0.3198; *p* = 0.0003) compared to penumbra volume (*p* = 0.01). Additionally, in the low-frequency range (0.07–0.20 Hz), CBFV and BP were inversely related to the gain, an index of vascular tone. Conclusion: CTP metrics appear to correlate with the outcome-relevant VLF phase and reactive hyperemic CBFV, which interact with BP to influence vascular tone and gain. These aspects of dCA could potentially guide BP management in patients with acute stroke undergoing recanalization therapy. However, further validation is required.

## 1. Introduction

Cranial computed tomography perfusion (CTP) is frequently used as a part of a multimodal imaging approach to evaluate patients with acute ischemic stroke (AIS). CTP is designed to differentiate between ischemic tissue at risk of brain cell death (penumbra) and the ischemic infarct core, where tissue damage is already irreversible. By applying specific clinical, time-dependent, and CTP criteria to identify a relevant mismatch between the infarct core or the clinical severity and the penumbral tissue, early recanalizing therapy, such as intravenous thrombolysis (IV lysis) and/or mechanical thrombectomy (MT), within 24 h after the first AIS symptom onset can salvage the tissue at risk, leading to improved clinical outcomes [1,2,3,4,5].

Early CTP also provides prognostic information, such as predicting modified Rankin Scale (mRs) outcomes based on core infarct volume [6,7], and assessing the impact of recanalization therapy on reducing brain edema and improving mRs [8,9]. Additionally, CTP can estimate the likelihood of post-interventional hyperemia [10,11,12]. Ischemia impairs cerebral autoregulation (CA), limiting the ability of the brain to regulate the blood supply, which can exacerbate ischemic damage under conditions of low blood pressure [13,14,15,16,17]. Because the CA, as assessed through dynamic cerebral autoregulation (dCA), does not immediately recover to full strength after vessel reopening, a factor of prognostic relevance [18,19], it seems reasonable to estimate the post-interventional state of (d)CA as early as possible. This knowledge can guide BP management. However, it is currently unknown whether CTP parameters can predict post-interventional dCA. We hypothesized that the volume of the penumbral or core infarct predicts dCA impairment in patients undergoing recanalization therapy. If this is the case, CTP could serve as the starting point for (d) CA-driven BP management, incorporating repeated easy-to-perform bedside dCA assessments to adjust BP levels according to the severity of dCA impairment [17].

## 2. Methods

This retrospective cohort study of patients with AIS was approved by the Ethics Committee of Northwest and Central Switzerland (EKNZ; 2024–01039). This study was conducted in accordance with the Declaration of Helsinki, adhered to good clinical practice standards, and was part of a larger trial registered at https://clinicaltrials.gov/ NCT04611672, dated 12 October 2020. At our institution, all patients or their relatives provide general consent, allowing the use of routinely acquired data for retrospective studies. Data supporting the findings of this study are available from the corresponding author upon reasonable request.

### 2.1. Study Setting

The stroke care protocols at our institution have been previously reported in detail [20,21].

**Figure 1:**
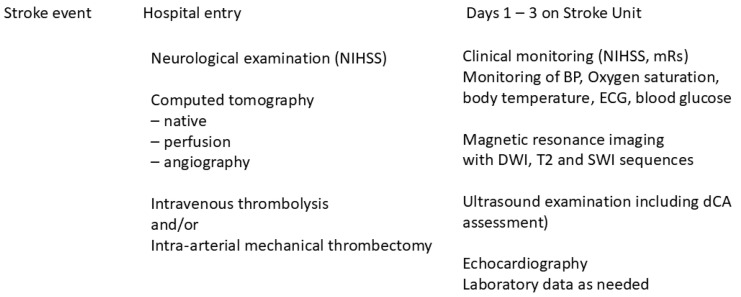
Flow chart of the patients’ disease/hospitalization course during the first days following the stroke event and their relation to the timing of diagnostic procedures for clinical data collection.

To summarize, all patients with stroke syndrome receive standardized care, with an initial focused clinical examination followed by multimodal cranial computed tomography (CT; Siemens Force, Edge, or XCeed CT, Siemens Healthineers, Forchheim, Germany). This includes non-enhanced cranial CT (NCCT), CTP postprocessed by Syngo.via (Siemens Healthineers) and Rapid CTP (RapidAI, San Mateo, CA, USA) to estimate the infarct core and penumbra, and CT angiography (CTA). If indicated, IV-lysis and/or MT are performed immediately. All patients diagnosed with stroke were transferred to the stroke unit for intensive clinical monitoring. This includes the National Institute of Health Stroke Scale (NIHSS) [22] and mRs [23] assessments upon hospital admission, as well as daily assessments while in the stroke unit and three months after the ischemic event (as the outcome measure). Blood pressure, heart rate, body temperature, blood glucose level, and oxygen saturation were closely monitored. Ultrasound examinations of all brain-supplying arteries, including dCA assessment, echocardiography, and brain magnetic resonance imaging (MRI) with diffusion-weighted imaging (DWI), T2, and susceptibility-weighted imaging (SWI) sequences, are performed within 72 h of hospitalization. Imaging is conducted on either a Siemens Vida fit (3 Tesla), Siemens Aera (1.5 Tesla), or Philips Achieva (3 Tesla) (Siemens Healthineers). After all relevant information is gathered, stroke etiology was classified based on the Trial of ORG 10172 in Acute Stroke Treatment (TOAST) classification [24]. Figure 1 provides an overview of the time-dependent data collection process.

### 2.2. Cranial Computed Tomography Perfusion (CTP)

Imaging was performed using Siemens devices (Siemens Healthineers), with detector rows ranging from 96 to 128. NCCT helical scans were acquired from the skull base to the vertex with the following scan parameters: 120 kV, 240 mA (with dose modulation when applicable), 0.6 mm collimation, 30.7 mm/s table speed, and 30.7 mm table feed per rotation. A CTA of the arterial cervical and intracranial vessels (from the aortic arch to the vertex) was performed with a prep scan at the level of the aortic arch to optimize timing of the arterial phase. A weight-adapted volume of contrast medium (1840 mg/s iodine, flow rate 6 mL/s), 100 kV, dose modulation, 0.6 mm collimation, 38.4 mm/s table speed, and 42.2. mm of table feed per rotation. CTP imaging was performed with a coverage of 10–11 cm in the Z axis, 60–65 s of total acquisition duration (one scan every 1.5 s), and 5 mm slice thickness. Parameters included 70 kV, 140 to 150 mA, 1.2 mm collimation, 38.4 mm/s table speed, 42.2 mm table feed per rotation, a weight-adapted volume of contrast medium (2240 mg/s iodine), and a recommended flow rate of 6 mL/s. After post-processing using Syngo.via and RapidAI, the tissue at risk (penumbra) was defined as having a time to maximum residue function (Tmax) prolongation of more than 6 s. Core infarction was defined as tissue with a relative cerebral blood flow <30% of the values obtained from the contralateral hemisphere.

### 2.3. Dynamic Cerebral Autoregulation (dCA)

**Figure 2:**
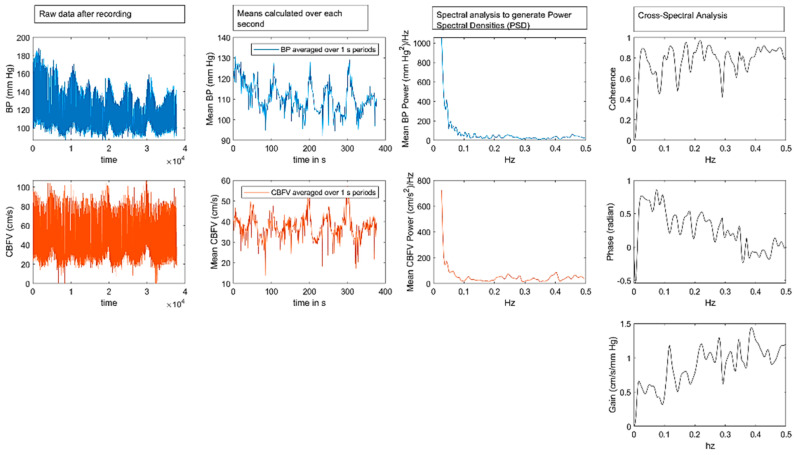
Illustration of performing dynamic cerebral autoregulation assessment. The first two images show the envelope curves of the blood pressure (BP) and cerebral blood flow velocity (CBFV) recordings. The data points of these time series are averaged over 1 s intervals to create new time series. From this, power spectra are generated. Cross-spectral analysis then extracts coherence, phase, and gain across the frequency range of 0–0.5 Hz. Despite some undulations, coherence is high, the phase decreases, and the gain increases. For reporting, the measured values are averaged over the following three frequency ranges: 0.02–0.07 Hz, 0.07–0.2 Hz, and 0.2–0.5 Hz. The transfer function model of dynamic cerebral autoregulation (dCA) reflects a high-pass filter behavior, that is, BP changes with a frequency of >0.2 Hz pass are transmitted through to CBFV; the exact physiological correlates in the lower frequency ranges (<0.20 Hz) are only partially understood. For example, the CO_2_ regulation is primarily observed in the 0.07–0.20 Hz range, while CBFV changes in the 0.02–0.07 Hz range (corresponding to blood flow changes every 20–50 s) may reflect blood volume changes in the microcirculation [26].

At our institution, dCA is routinely included in the transcranial Doppler sonographic evaluation of patients with suspected ischemic cerebrovascular impairment to evaluate CA function. All investigations were performed with the patient in the supine position, with the head elevated by approximately 30°. Middle cerebral artery (MCA) cerebral blood flow velocity (CBFV) was recorded using a 2 MHz probe (MultidopX, DWL; Compumedics, Sipplingen, Germany), and blood pressure (BP) was measured simultaneously using a Finometer Pro (Finapres Medical Systems, Amsterdam, The Netherlands) for a minimum of 6 min. End-tidal pCO_2_ (ETCO_2_) concentration was measured using nostril tubes and a capnograph embedded in a TCD device. BP, CBFV, and ETCO_2_ data were collected at a frequency of 100 Hz. Data were analyzed using MATLAB (version 2023a; MathWorks Inc., Natick, MA, USA). The data were visually inspected for artifacts, and only artifact-free periods of 5 min were used for the analysis. Each raw data time series was averaged over 1 s intervals to generate new time series representing the mean CBFV and BP. Coherence, phase, and gain between the BP and CBFV time series were extracted using their respective power auto-spectra or cross-spectra with Welch’s averaged period gram method. A Hanning window with a length of 100 s, a window with a 50% overlap, and a total fast Fourier transformation data length of 300 s were used. For each patient, coherence, phase (in radians), and gain (in cm/s/mmHg) were calculated over a frequency range of 0.02–0.50 Hz. These values were then averaged within three frequency bands, namely, very low frequency range (VLF, 0.02–0.07 Hz), low frequency range (LF, 0.07–0.20 Hz), and high frequency range (HF, 0.2–0.5 Hz) [25,26]. The gain and phase usually follow opposite directions, that is, low gain is associated with high phase, and vice versa. Impaired dCA is indicated by a lower phase or higher gain in either the VLF or LF range. An illustration of this in investigation is presented in Figure 2.

### 2.4. Patients

This study included consecutive patients treated in our stroke unit between 1 January 2020 and 31 April 2022. Patients who underwent dCA assessments were retrospectively reviewed. The inclusion criteria for this study were ≥18 years of age, absence of pregnancy, diagnosis of a characteristic hemispheric syndrome, confirmed as a definitive supratentorial ischemic stroke in the MCA territory through initial multimodal imaging and later validated by DWI imaging, and high-quality bilateral dCA assessments (stroke-affected hemisphere (AH); stroke-unaffected hemisphere (UH)). Exclusion criteria included the final diagnosis of a stroke mimic, a primary intracranial hemorrhage, a transient ischemic attack, and a cerebral sinus or vein thrombosis.

Results are reported separately for the AH and UH to account for hemispheric differences in dCA function.

### 2.5. Statistical Analyses

All data analyses were performed using the MATLAB Statistical Toolbox (MathWorks Inc.). Data are presented as mean ± standard deviation (SD) for normally distributed variables and as median with their interquartile range (IQR) for non-normally distributed variables. Most continuous variables were not normally distributed; therefore, we used the nonparametric Kruskal–Wallis test for between-group comparisons. Fisher’s exact test or chi-square statistics were applied for comparing categorical variables. Univariate and multiple linear regression analyses were performed to determine whether CTP-derived penumbra or infarct core volume could predict dCA parameters. Baseline variables that are reported to influence dCA, including age, history of arterial hypertension, cardiac left ventricular ejection fraction (LVEF%), CBFV, BP, and ETCO_2_ (averaged over the dCA recording period), were included together with penumbra and infarct core volume. If either penumbra or infarct core volume, along with one or more baseline variables, were significantly related to dCA parameters, multiple linear regression models were constructed to further evaluate the role of these parameters. To address collinearity between penumbra and infarct core volume—representing different stages of cerebral ischemia—two separate multiple regression models were created. The first model included penumbra volume only, and the second included infarct core volume only [27]. Statistical significance was set at *p* < 0.05.

## 3. Results

A total of 336 patients (median age, 71 (59–78) years; 102 women (30.3%)) meeting the inclusion criteria were analyzed. Of them, 165 patients (age 73 (60–80) years; 43 women (26%)) had undergone recanalizing therapy, with 91 patients receiving IV-lysis alone and 74 undergoing MT. Stroke etiology was classified according to TOAST into cardio-embolism (*n* = 64), atherosclerotic large vessel disease (*n* = 35), lacunar stroke (*n* = 17), other defined sources (such as dissections; *n* = 10), and embolic strokes of unknown sources (*n* = 39). Other baseline characteristics are presented in Table 1. We focused our analysis on IV lysis and MT as recanalizing therapies without further subclassifying the patients with MT into those who received bridging IV lysis therapy and those who did not.

### 3.1. MT Procedures

Of the 74 patients, 47 received bridging therapy with prior IV lysis. Intracranial thrombectomy was exclusively performed using stent retriever systems. Vasospasm, a complication of MT, occurred in six instances. After MT, 31 patients achieved complete recanalization (thrombolysis in cerebral infarction (TICI) 3), while 43 had varying degrees of incomplete recanalization (TICI 2a, *n* = 7; 2b, *n* = 18; 2c, *n* = 18) [28]. If an extracranial carotid artery stenosis was ≥50% according to NASCET [29] or a carotid occlusion was the cause of the stroke and this artery obstruction was traversable for thrombectomy maneuver, these obstructions were permanently recanalized by stent placement.

Patients undergoing MT had a larger median penumbra and infarct core (on both CTP and MRI) and exhibited more severe neurological impairment at hospital admission and at 3 months post-stroke.

### 3.2. Dynamic Cerebral Autoregulation (dCA) Assessment

The earliest dCA assessment post-stroke onset was performed at varying times in 114 patients within the first 24 h after, 37 patients between 24 and 48 h, and 14 patients between 48 and 72 h. The minimum and maximum mean BP over the recording periods were 57 mmHg and 128 mmHg, respectively. In the affected hemisphere (Table 2), CBFV, VLF, and LF gains were significantly higher in patients who underwent MT than in those who underwent IV lysis. Conversely, the VLF and LF phases were significantly lower in the MT group. In the unaffected hemisphere, only the gain and phase in the LF range differed between the two groups.

### 3.3. Linear Regression Analysis

#### 3.3.1. CBFV in the Affected Hemisphere

**Figure 3:**
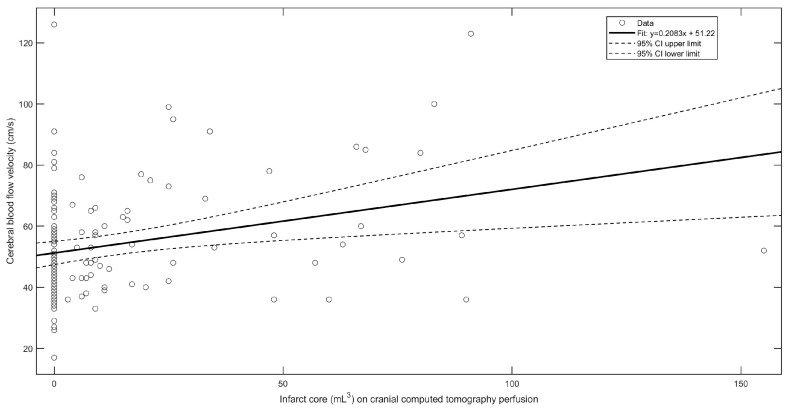
Linear regression model of cerebral blood flow velocity and ischemic infarct core volume on cranial computed tomography perfusion.

In patients treated with MT, the CBVF in the AH was significantly higher compared to both patients receiving IV lysis alone and UH. Univariate linear regression analysis (Table 3) revealed that CBFV in the AH was significantly associated with penumbra size (*p* = 0.01) and core infarct volume (*p* = 0.003; Figure 3). These findings suggest that increased CBFV in the AH corresponds to reactive hyperemic blood flow.

#### 3.3.2. VLF Phase in the Affected Hemisphere

In the univariate linear regression analysis (Table 4), it was observed that the CBFV, penumbra, and infarct core volume were significantly related to the VLF phase.

**Figure 4:**
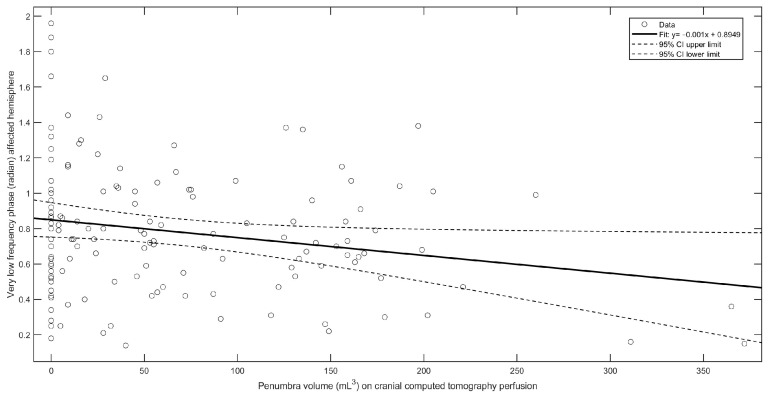
Linear regression model of very low frequency phase and ischemic penumbra on cranial computed tomography perfusion.

In the multiple regression model with CBFV and penumbra or infarct core volume, the infarct core volume (*p* = 0.01) and penumbra volume (*p* = 0.004; Figure 4) remained significantly related to the VLF phase in the AH.

#### 3.3.3. LF Phase in the Affected Hemisphere

Univariate linear regression analysis (Table 4) identified significant associations between LF phase in the AH and several independent variables, including CBFV, LVEF, mean BP, and mean ETCO_2_ over the recording period, age, arterial hypertension, penumbra size, and core infarct volume. In the multiple linear regression models with mean BP over the recording period and penumbra as the independent variables, the LF phase in the AH was significantly associated with penumbra (*p* = 0.04) and showed a trend-level association with mean BP (*p* = 0.06).

#### 3.3.4. VLF Gain in the Affected Hemisphere

Univariate linear regression analysis (Table 5) revealed a significant association between VLF gain in the AH and several independent variables, including CBFV, LVEF, mean BP, and mean ETCO_2_ over the recording period, age, arterial hypertension, and penumbra or core infarct volume. In the various multiple linear regression analysis models with CBFV, mean BP over the recording period, and either penumbra or infarct core volume as independent variables, only CBFV remained significantly related to VLF gain in the AH (*p* = 0.0000).

#### 3.3.5. LF Gain in the Affected Hemisphere

Univariate linear regression analysis (Table 5) revealed significant associations between LF gain in the AH and several independent variables, including CBFV, LVEF, and mean BP, whereas penumbra and infarct core volume did not show any association. In the multiple linear regression model with CBFV, LVEF, and mean BP over the recording period as the independent variables and LF gain in the AH as the dependent variable, CBFV (*p* = 0.0000) and mean BP over the recording period (*p* = 0.0000) remained significantly related to LF gain.

#### 3.3.6. Unaffected Hemisphere

In the unaffected hemisphere, the VLF phase was not associated with any of the predefined independent variables (Table 6).

The LF phase was related to mean BP over the recording period only (*p* = 0.0002, Table 7).

In univariate linear regression analysis, VLF gain was related to mean BP over the recording period, infarct core volume, and age (Table 8).

In the multiple linear regression model, mean BP over the recording period, age, and infarct core volume were the independent variables, and VLF gain in the UH was the dependent variable. Age (*p* = 0.03) and mean BP over the recording period (*p* = 0.03) remained significantly related to VLF gain in the UH.

Univariate linear regression analysis revealed that LF gain in the UH was significantly related to CBFV, mean BP over the recording period, infarct core volume, and penumbra, with LVEF showing a trend-level relationship (Table 9). In the multiple linear regression models with CBFV, mean BP over the recording period, and either penumbra or infarct core volume as the independent variables and LF gain in the UH as the dependent variable, mean BP (*p* = 0.003), CBFV (*p* = 0.008), core (*p* = 0.01), and penumbra (*p* = 0.01) remained significantly related to LF gain in the UH.

## 4. Discussion

The two patient groups that received recanalization therapy (IV lysis vs. MT) exhibited notable differences. These clinical and CTP examination differences are most likely due to the grouping of our patients into those without a large vessel occlusion who received IV lysis only and those with large vessel occlusion who underwent MT, regardless of whether IV lysis was performed prior to MT. Regarding the cerebral hemodynamic responses, the IV lysis group exhibited, on average, more favorable outcomes, with higher VLF and LF phases and lower VLF and LF gain values in the AH. These findings are likely attributable to the absence of a large vessel occlusion in this group.

Overall, our main findings indicate that early CTP-detected ischemic parameters are associated with the future cerebral hemodynamic parameter, including phase, blood flow velocity, and gain.

When the VLF phase was assessed within the first 72 h after a stroke, a higher phase was associated with better outcomes, as defined by an mRs score < 2, and vice versa [19]. Our results indicated that the predicted VLF phase values increased as the volume of ischemic tissue (both penumbra and infarct core) decreased. This inverse relationship aligns with previous reports indicating that the dCA is more disrupted with larger brain infarct volumes [18,30]. Although the relationship observed in our patient cohort was not very strong, this study provides the first evidence that early CTP-derived ischemic parameters are associated with the dCA state in the affected hemisphere within the 48–72 h.

In the affected AH, VLF gain was related to CBFV, age, and mean BP over the recording period in the UH. In AH, it is plausible to assume that the elevated CBFV values in thrombectomy patients corresponded to a reactive hyperemic CBF that increased vascular gain (positively directed β-coefficient). In the UH group, VLF gain decreased with age, which is a noteworthy finding, as it contradicts trends observed in healthy, normal individuals [25,31]. Whether this observed age-related decrease in VLF gain represents a physiological response to a stroke event requires further research.

The LF phase in the AH was strongly associated with the penumbra and showed a trend towards significance with mean BP. The mean BP recorded for our patients was within the autoregulatory range usually considered normal of approximately 50–150 mm Hg [32]. Within this range, it is plausible that the LF phase varies, being lower at the lower end of BP and higher at the upper end. Recently, Wang and Payne [32] suggested that the CA may function adequately only within a narrow range of 80–100 mmHg of mean BP. Hence, a substantial number of our patients could have been at risk of secondary ischemia at the time of the dCA assessment. According to our results, the penumbra could be the first indicator of such a risk. In the UH, the LF phase showed a strong relationship with mean BP over the recording period but no association with CTP parameters (not even in the univariate analysis). Considering that LF phase is lower than our normative data (0.74 ± 0.20 radians) [25], further research is needed to clarify the clinical relevance of such lower phase values in the light of Wang and Payne’s findings.

In the AH, LF gain was positively correlated with CBFV, suggesting that hyperemic CBF contributes to increased LF gain. Mean BP was negatively related to LF gain, indicating that an increase in BP leads to a decrease in gain. This could be due to the greater vasodilation of ischemia-damaged vessels, resulting in decreased gain, counterbalanced by the gain-enhancing effects of hyperemic CBFV. In the UH, LF gain is positively correlated with ischemic challenge in the brain. The gain in the UH was also negatively correlated with BP, as in the AH. This is also noteworthy, as the similarity in the BP–gain relationship between the two hemispheres, combined with the dependency of LF gain on ischemic factors and reactive hyperemic blood flow, suggests that ischemia triggers analogous autoregulatory processes in both hemispheres at the LF range.

### Study Limitations

Although significant, the relatively low R^2^ values indicate that the explanatory power of early CTP on future cerebral hemodynamics is weak, suggesting that other factors must play a role. The major limitation of this retrospective study is that our analysis assumed stable conditions between the data collected at different time points or days, disregarding potential dynamic changes, such as the initiation of restorative processes, which would have caused early dCA recovery. Another limitation is the BP dependency of dCA, that is, post-interventional BP management and the natural decline in autonomic stimulation after the acute phase of a stroke or therapeutic interventions could significantly alter dCA. Additionally, cardiac output and systemic vascular resistance may change over time because they depend on the autonomic state of patients. Thus, the reasons for the low R^2^ values are not clear, and additional studies are required to determine the factors that were not measured in this study. Furthermore, we discuss whether standardized time points of dCA assessments would increase the predictive power. In our analysis, the following three clinically different patient groups were combined: those with IV-lysis only, those with MT only, and those who received IV-lysis and MT. The outcomes of dCA, especially in the IV-lysis/MT group, may not be the only effect of IV-lysis plus MT. When analyzing the relationship between ischemic tissue (e.g., the infarct core and penumbra) and VLF phase separated for each group, these relationships were no longer significant, underscoring the impact of sample size and variability. Finally, dCA assessment techniques are vulnerable to intraindividual differences [33,34,35].

Another limiting factor in our study was the use of CTP [27,36,37]. Cardiac output and the exact timing of contrast agent application are the reasons for inter-individual differences that can be accentuated by the patient’s head movement. The latter also affects the data acquisition regions for the calculation of the penumbra and infarct core volume. Different CTP protocols may generate variations in results, hindering the rapid generalization of monocentric findings. The dCA evaluates the CBFV response to BP changes over 2–50 s, with the VLF examining time periods between 15 and 50 s. The CTP data acquisition time used to calculate cerebral blood volume spans 20–40 s after contrast agent application [27] or up to 60–90 s (our protocol and Fainardi et al. [33]). This raises the question of whether dCA in the VLF is more likely to investigate pressure-dependent CBV changes or CBF changes contaminated by volume changes.

## 5. Conclusions

The assessment of cerebral autoregulation using dynamic cerebral autoregulation (dCA) can predict patient outcomes within 72 h after a stroke event [19]. In this retrospective study, we identified, for the first time, a weak but notable association between the volume of the ischemic tissue, as determined by the initial CTP to predict dCA impairment, and the occurrence of reactive hyperemic CBFV. Along with BP, CBFV determines vascular tone, which might be important for secondary hemorrhage. However, before these findings can translate into clinical applications, additional research is necessary to confirm and improve the predictive ability of CTP.

## Figures and Tables

**Table 1:** Clinical baseline characteristics, risk factors, and computed tomography perfusion results in the acute ischemic stroke patients having undergone recanalizing therapy dichotomized into those who had received intravenous thrombolysis (IV-lysis) only, versus those who had undergone mechanical thrombectomy (MT) with or without prior bridging IV-lysis.

	IV-Lysis Only (*n* = 91)	MT Procedure (*n* = 74)	*p*-Value
Age (years)	71.5 (58–78)	75 (63–82)	0.16
Women/men	20/71	24/50	0.19
NIHSS at entry	5 (3–7)	15 (9–18)	0.0000
mRs at 3 months	1 (0–2)	3 (1–3)	0.0000
mRs at 3 months			
dichotomized			
≤2	75	36	0.0000
>2	16	38	
Arterial hypertension (*n*)	50	43	0.59
Diabetes mellitus (*n*)	25	20	1
Dyslipidemia (*n*)	64	60	0.72
Left ventricular ejection fraction (%)	59 (55–63)	58 (50–65)	0.57
Ischemic heart disease (*n*)	19	29	0.30
Estimated glomerular filtration rate (mL/min/10.72 m^2^)	820.5 (67–91)	79 (59–89)	0.20
Blood pressure (mm Hg) at admission			
Systolic	153 (137–172)	147 (134–164)	0.10
Mean	106 (93–117)	103 (91–113)	0.22
Diastolic	85 (74–96)	81 (71–91)	0.12
Neuroimaging			
CTP—Penumbra (mL)	5 (0–28)	125 (55–162)	0.0000
CTP—Core (mL)	0 (0–0)	9 (0–34)	0.0000
MRI—infarct volume (mL) on DWI	20.8 (00.54–110.5)	150.3 (60.4–500.9)	0.0000

CTP, computed tomography; DWI, diffusion-weighted imaging; MRI, magnetic resonance imaging; mRs, modified Rankin scale; NIHSS, National Institutes of Health Stroke Scale. Values are presented as either numbers or medians and the interquartile range (IQR).

**Table 2:** Comparison of transfer function estimates after recanalization in the middle cerebral artery of the stroke-affected hemisphere between the patients who had received intravenous thrombolysis (IV-lysis) alone versus patients who had undergone the mechanical thrombectomy (MT) procedure.

	IV-Lysis Only (*n* = 91)	MT Procedures (*n* = 74)	*p*-Value
Mean arterial blood pressure (mm Hg) over recording period	98 (86–104)	89 (79–102)	0.06
Heart rate	68 (60–80)	70 (62–79)	0.41
ETCO_2_ (mm Hg) over recording period	39.5 (38.1–40.5)	39.2 (37.6–40.8)	0.40
Affected Hemisphere			
CBFV (cm/s)	46 (41–55)	57 (48–75)	0.0000
Pulsatility index	0.95 (0.85–1.12)	0.98 (0.86–1.08)	0.45
Coherence			
VLF	0.57 (0.48–0.64)	0.62 (0.53–0.70)	0.04
LF	0.63 (0.53–0.77)	0.66 (0.53–0.78)	0.92
HF	0.68 (0.58–0.76)	0.64 (0.55–0.70)	0.15
Gain (mmHg/cm/s)			
VLF	0.25 (0.15–0.38)	0.36 (0.23–0.55	0.001
LF	0.39 (0.28–0.52)	0.49 (0.32–0.66)	0.01
HF	0.51 (0.38–0.64)	0.56 (0.40–0.86)	0.06
Phase (radian)			
VLF	0.83 (0.59–1.06)	0.67 (0.46–0.85)	0.002
LF	0.68 (0.50–0.91)	0.53 (0.36–0.76)	0.001
HF	0.25 (0.06–0.49)	0.19 (0.03–0.39)	0.16
Unaffected Hemisphere			
CBFV (cm/s)	47 (41–56)	49 (42–50)	0.32
Pulsatility Index	0.93 (0.84–1.11)	0.97 (0.85–1.10)	0.46
Coherence			
VLF	0.57 (0.50–0.65)	0.59 (0.48–0.67)	0.95
LF	0.62 (0.52–0.75)	0.61 (0.52–0.76)	0.71
HF	0.62 (0.52–0.77)	0.64 (0.49–0.72)	0.77
Gain (mm Hg/cm/s)			
VLF	0.26 (0.15–0.41)	0.33 (0.17–0.48)	0.17
LF	0.44 (0.26–0.56)	0.55 (0.37–0.77)	0.01
HF	0.48 (0.38–0.68)	0.62 (0.47–0.80)	0.01
Phase (radian)			
VLF	0.80 (0.61–1.04)	0.82 (0.52–1.08)	0.86
LF	0.64 (0.49–0.81)	0.56 (0.39–0.76)	0.045
HF	0.27 (0.04–0.48)	0.19 (0–0.32)	0.13

CBFV, cerebral blood flow velocity; ETCO_2_, end-tidal carbon dioxide; HF, high frequency; LF, low frequency; VLF, very low frequency.

**Table 3:** Univariate linear regression to predict cerebral blood flow velocity in the middle cerebral artery of the affected hemisphere.

Variable	
Left ventricular ejection fraction (LVEF, %)	*β* = 0.019 (95%CI −0.2202 to 0.2583), r^2^ = 0.0002, F(2,163) = 0.02, *p* = 0.80
Mean BP (mm Hg) over the recording period	*β* = −0.116 (95%CI −0.3001 to 0.0664), r^2^ = 0.009, F(2,163) = 1.58, *p* = 0.20
Mean ETCO_2_ (mm Hg) over the recording period	*β* = −0.044 (95%CI −1.0060 to 0.9163), r^2^ = 0.001, F(2,163) = 0.008, *p* = 0.92
Penumbra (mL^3^)	*β* = 0.047 (95%CI 0.0108 to 0.0840), r^2^ = 0.043, (F(2,163) = 6.54, *p* = 0.01
Core (mL^3^)	*β* = 0.208 (95%CI 0.0968 to 0.3198), r^2^ = 0.085, F(2,163) = 13.64, *p* = 0.0003
Age (years)	*β* = −0.168 (95%CI −0.3447 to 0.00859), r^2^ = 0.021, F(2,163) = 3.53, *p* = 0.06
Arterial hypertension	*β* = −3.063 (95%CI −8.4308 to 2.3030), r^2^= 0.007, F(2,163) = 1.27, *p* = 0.26

*β*, beta coefficient; BP, blood pressure; ETCO_2_, end-tidal carbon dioxide.

**Table 4:** Univariate linear regression analysis to predict very low-frequency and low-frequency phases in the middle cerebral artery of the affected hemisphere.

Variable	Very Low Frequency Range
CBFV (cm/s)	*β* = −0.004 (95%CI −0.0074 to −0.0008), r^2^ = 0.03, F(2,163) = 5.98, *p* = 0.01
LVEF (%)	*β* = 0.0032 (95%CI −0.0021 to 0.0084), r^2^ = 0.009, F(2,163) = 1.43, *p* = 0.23
Mean BP (mm Hg) over recording period	*β* = 0.002 (95%CI −0.0021 to 0.0060), r^2^ = 0.005, F(2,163) = 0.88, *p* = 0.34
Mean ETCO_2_ (mm Hg) over recording period	*β* = 0.003 (95%CI −0.0176 to 0.0241), r^2^ = 0.0007, F(2,163) = 0.09, *p* = 0.75
Penumbra (mL^3^)	*β* = −0.001 (95%CI −0.0018 to −0.0002), r^2^ = 0.042, F(2,163) = 6.76, *p* = 0.01
Core (mL^3^)	*β* = −0.003 (95%CI −0.0054 to −0.0005), r^2^ = 0.038, F(2,163) = 5.62, *p* = 0.01
Age (years)	*β* = 0.0002 (95%CI −0.0037 to 0.0041), r^2^ = 0.0001, F(2,163) = 0.009, *p* = 0.92
Arterial hypertension	*β* = 0.05 (95%CI −0.0653 to 0.1713), r^2^ = 0.005, F(2,163) = 0.78, *p* = 0.37
Variable	Low Frequency Range
CBFV (cm/s)	*β* = −0.005 (95%CI −0.0058 to 0.0002), r^2^ = 0.02, F(2,163) = 3.30, *p* = 0.07
LVEF (%)	*β* = 0.004 (95%CI −0.0001 to 0.0089), r^2^ = 0.023, F(2,163) = 3.67, *p* = 0.06
Mean BP (mm Hg) over recording period	*β* = 0.004 (95%CI 0.0004 to 0.0075), r^2^ = 0.029, F(2,163) = 4.80, *p* = 0.02
Mean ETCO_2_ (mm Hg) over recording period	*β* = 0.015 (95%CI −0.0011 to 0.0329), r^2^ = 0.022, F(2,163) = 3.39, *p* = 0.06
Penumbra (mL^3^)	*β* = −0.0009 (95%CI −0.0016 to −0.0002), r^2^ = 0.040, F(2,163) = 6.01, *p* = 0.01
Core (mL^3^)	*β* = −0.0008 (95%CI −0.0030 to 0.0014), r^2^ = 0.003, F(2,163) = 0.55, *p* = 0.45
Age (years)	*β* = 0.001 (95%CI −0.0018 to 0.0051), r^2^ = 0.005, F(2,163) = 0.86, *p* = 0.35
Arterial hypertension	*β* = 0.051 (95%CI −0.0503 to 0.1591), r^2^ = 0.006, F(2,163) = 1.05, *p* = 0.30

*β*, beta coefficient; BP, blood pressure; CBFV, cerebral blood flow velocity; ETCO_2_, end-tidal carbon dioxide; LVEF, left ventricular ejection fraction.

**Table 5:** Univariate linear regression analysis to predict very low-frequency and low-frequency gain in the middle cerebral artery of the affected hemisphere.

Variable	Very Low Frequency Range
CBFV (cm/s)	*β* = 0.006 (95%CI 0.0050 to 0.0087), r^2^ = 0.24, F(2,163) = 51.64, *p* = 0.0000
LVEF (%)	*β* = −0.001 (95%CI −0.0050 to 0.0018), r^2^ = 0.006, F(2,163) = 0.91, *p* = 0.34
Mean BP (mm Hg) over recoding period	*β* = −0.002 (95%CI −0.0052 to −0.0000), r^2^ = 0.02, F(2,163) = 3.97, *p* = 0.04
Mean ETCO_2_ (mm Hg) over recording period	*β* = 0.002 (95%CI −0.0112 to 0.0156), r^2^ = 0.007, F(2,163) = 0.10, *p* = 0.74
Penumbra (mL^3^)	*β* = 0.0005 (95%CI 0.0000 to 0.0010), r^2^ = 0.02, F(2,163) = 3.94, *p* = 0.04
Core (mL^3^)	*β* = 0.002 (95%CI 0.0011 to 0.0041), r^2^ = 0.07, F(2,163) = 11.73, *p* = 0.0008
Age (years)	*β* = −0.001 (95%CI −0.0040 to 0.0010), r^2^ = 0.008, F(2,163) = 1.38, *p* = 0.24
Arterial hypertension	*β* = −0.02 (95%CI −0.1006 to 0.0508), r^2^ = 0.002, F(2,163) = 0.42, *p* = 0.51
Variable	Low frequency range
CBFV (cm/s)	*β* = 0.003 (95%CI 0.0034 to 0.0074), r^2^ = 0.15, (F(2,163) = 29.05, *p* = 0.0000
LVEF (%)	*β* = −0.003 (95%CI −0.0065 to 0.0000), r^2^ = 0.02, F(2,163) = 3.84, *p* = 0.05
Mean BP (mm Hg) over recording period	*β* = −0.004 (95%CI −0.0073 to −0.0024), r^2^ = 0.08, F(2,163) = 15.42, *p* = 0.0001
Mean ETCO_2_ (mm Hg) over recording period	*β* = −0.01 (95%CI −0.0234 to 0.0026), r^2^ = 0.01, F(2,163) = 2.51, *p* = 0.11
Penumbra (mL^3^)	*β* = 0.0004 (95%CI −0.0001 to 0.0008), r^2^ = 0.01, F(2,163) = 2.16, *p* = 0.14
Core (mL^3^)	*β* = 0.0001 (95%CI −0.0014 to 0.0016), r^2^ = 0.0001, F(2,163) = 0.01, *p* = 0.90
Age (years)	*β* = 0.001 (95%CI −0.0014 to 0.0036), r^2^ = 0.005, F(2,163) = 0.79, *p* = 0.37
Hypertension	*β* = 0.006 (95%CI −0.0678 to 0.0807), r^2^ = 0.002, F(2,163) = 0.02, *p* = 0.86

*β*: beta coefficient; BP: blood pressure; CBFV: cerebral blood flow velocity; ETCO_2_: end-tidal carbon dioxide; LVEF: left ventricular ejection fraction.

**Table 6:** Univariate linear regression analysis to predict very low-frequency phase in the middle cerebral artery of the unaffected hemisphere.

Variable	
CBFV (cm/s)	*β* = −0.002 (95%CI −0.0061 to 0.0018), r^2^ = 0.009, F(2,163) = 1.13, *p* = 0.24
LVEF (%)	*β* = 0.002 (95%CI −0.0036 to 0.0088), r^2^ = 0.005, F(2,163) = 0.66, *p* = 0.41
Mean BP (mm Hg) over recording period	*β* = 0.003 (95%CI −0.0010 to 0.0085), r^2^ = 0.01, F(2,163) = 2.49, *p* = 0.11
Mean ETCO_2_ (mm Hg) over recording period	*β* = −0.009 (95%CI −0.0362 to 0.0164), r^2^ = 0.004, F(2,163) = 0.55, *p* = 0.45
Penumbra (mL^3^)	*β* = 0.000 (95%CI −0.001 to 0.001), r^2^ = 0.000, F(2,163) = 0.000, *p* = 0.98
Core (mL^3^)	*β* = −0.001 (95%CI −0.0044 to 0.0016), r^2^ = 0.007, F (2,72) = 0.87, *p* = 0.35
Age (years)	*β* = 0.0004 (95%CI −0.0041 to 0.0048), r^2^ = 0.0002, F (2,72) = 0.02, *p* = 0.86
Arterial hypertension	*β* = −0.026 (95%CI −0.1646 to 0.1111), r^2^ = 0.001, F (2,72) = 0.14, *p* = 0.70

*β*, beta coefficient; BP, blood pressure; CBFV, cerebral blood flow velocity; ETCO_2_, end-tidal carbon dioxide; LVEF, left ventricular ejection fraction.

**Table 7:** Univariate linear regression analysis to predict low-frequency phase in the middle cerebral artery of the unaffected hemisphere.

Variable	
CBFV (cm/s)	*β* = −0.006 (95%CI −0.0060 to 0.0010), r^2^ = 0.01, F(2,163) = 1.97, *p* = 0.16
LVEF (%)	*β* = 0.004 (95%CI −0.0009 to 0.0102), r^2^ = 0.02, F(2,163) = 2.78, *p* = 0.09
Mean BP (mm Hg) over recording period	*β* = 0.007 (95%CI 0.0038 to 0.0118), r^2^ = 0.10, F(2,163) = 14.8, *p* = 0.0002
Mean ETCO_2_ (mm Hg) over recording period	*β* = −0.001 (95%CI −0.0241 to 0.0221), r^2^ = 0.000, F(2,163) = 0.007, *p* = 0.93
Penumbra (mL^3^)	*β* = 0.000 (95%CI −0.0008 to 0.0009), r^2^ = 0.0001, F(2,163) = 0.006, *p* = 0.93
Core (mL^3^)	*β* = 0.001 (95%CI −0.0013 to 0.0039), r^2^ = 0.009, F(2,163) = 1.03, *p* = 0.31
Age (years)	*β* = 0.0004 (95%CI −0.0036 to 0.0043), r^2^ = 0.0003, F(2,163) = 0.03, *p* = 0.85
Arterial hypertension	*β* = 0.096 (95%CI −0.0244 to 0.2181), r^2^ = 0.01, F(2,163) = 2.49, *p* = 0.11

*β*, beta coefficient; BP, blood pressure; CBFV, cerebral blood flow velocity; ETCO_2_, end-tidal carbon dioxide; LVEF, left ventricular ejection fraction.

**Table 8:** Univariate linear regression analysis to predict very low-frequency gain in the middle cerebral artery of the unaffected hemisphere.

Variable	
CBFV (cm/s)	*β* = 0.002 (95%CI −0.0005 to 0.0049), r^2^ = 0.02, F(2,163) = 2.65, *p* = 0.10
LVEF (%)	*β* = −0.007 (95%CI −0.0051 to 0.0036), r^2^ = 0.000, F(2,163) = 0.11, *p* = 0.73
Mean BP (mm Hg) over recording period	*β* = −0.003 (95%CI −0.0064 to 0.0001), r^2^ = 0.02, F(2,163) = 3.71, *p* = 0.05
Mean ETCO_2_ (mm Hg) over recording period	*β* = −0.0003 (95%CI −0.0188 to 0.0182), r^2^ = 0.000, F(2,163) = 0.001, *p* = 0.97
Penumbra (mL^3^)	*β* = 0.0005 (95%CI −0.0002 to 0.0011), r^2^ = 0.01, F (2,72) = 2.00, *p* = 0.15
Core (mL^3^)	*β* = 0.002 (95%CI 0.0005 to 0.0044), r^2^ = 0.05, F(2,163) = 5.91, *p* = 0.01
Age (year)	*β* = −0.004 (95%CI −0.0072 to −0.0013), r^2^ = 0.06, F(2,163) = 8.07, *p* = 0.005
Arterial hypertension	*β* = −0.04 (95%CI −0.1424 to 0.0463), r^2^ = 0.008, F(2,163) = 1.01, *p* = 0.31

*β*, beta coefficient; BP, blood pressure; CBFV, cerebral blood flow velocity; ETCO_2_, end-tidal carbon dioxide; LVEF, left ventricular ejection fraction.

**Table 9:** Univariate linear regression analysis to predict low-frequency gain in the middle cerebral artery of the unaffected hemisphere.

Variable	
CBFV (cm/s)	*β* = 0.004 (95%CI 0.0012 to 0.0070), r^2^ = 0.06, F(2,163) = 7.92, *p* = 0.005
LVEF (%)	*β* = −0.004 (95%CI −0.0087 to 0.0004), r^2^ = 0.02, F(2,163) = 3.23, *p* = 0.07
Mean BP (mm Hg) over recording period	*β* = −0.005 (95%CI −0.0090 to −0.0022), r^2^ = 0.07, F(2,163) = 10.6, *p* = 0.001
Mean ETCO_2_ (mm Hg) over recording period	*β* = 0.0005 (95%CI −0.0188 to 0.0199), r^2^ = 0.000, F(2,163) = 0.003, *p* = 0.95
Penumbra (mL^3^)	*β* = 0.001 (95%CI 0.0004 to 0.0018), r^2^ = 0.07, F(2,163) = 9.37, *p* = 0.002
Core (mL^3^)	*β* = 0.003 (95%CI 0.0013 to 0.0057), r^2^ = 0.08, F(2,163) = 10.07, *p* = 0.001
Age (years)	*β* = −0.001 (95%CI −0.0044 to 0.0022), r^2^ = 0.003, F(2,163) = 0.44, *p* = 0.50
Arterial hypertension	*β* = 0.01 (95%CI −0.0863 to 0.1192), r^2^ = 0.008, F(2,163) = 0.10, *p* = 0.75

*β*, beta coefficient; BP, blood pressure; CBFV, cerebral blood flow velocity; ETCO_2_, end-tidal carbon dioxide; LVEF, left ventricular ejection fraction.

## Data Availability

The corresponding author can provide all data on reasonable request.

## Abbreviations

AIS	acute ischemic stroke
BP	arterial blood pressure
CBFV	cerebral blood flow velocity in the middle cerebral artery
CTP	computed tomography perfusion
NIHSS	National Institute of Health Stroke Scale
mRs	modified Rankin scale
MT	mechanical thrombectomy
MRI	magnetic resonance imaging with diffusion-weighted imaging (DWI); T2 sequences and susceptibility-weighted imaging (SWI)
IQR	interquartile range
ETCO_2_	end-tidal carbon dioxide tension
LVEF (%)	left ventricular ejection fraction (percentage)
VLF	very low frequencies (0.02–0.07 Hz)
LF	low frequencies (0.07–0.20 Hz)
HF	high frequencies (>0.20–0.50 Hz)
